# Copy number variation of human *AMY1* is a minor contributor to variation in salivary amylase expression and activity

**DOI:** 10.1186/s40246-017-0097-3

**Published:** 2017-02-20

**Authors:** Danielle Carpenter, Laura M. Mitchell, John A. L. Armour

**Affiliations:** School of Life Sciences, University of Nottingham, Nottingham, NG7 2UH UK

**Keywords:** Genome instability, Amylase, CNV, Gene expression

## Abstract

**Background:**

Salivary amylase in humans is encoded by the copy variable gene *AMY1* in the amylase gene cluster on chromosome 1. Although the role of salivary amylase is well established, the consequences of the copy number variation (CNV) at *AMY1* on salivary amylase protein production are less well understood. The amylase gene cluster is highly structured with a fundamental difference between odd and even *AMY1* copy number haplotypes. In this study, we aimed to explore, in samples from 119 unrelated individuals, not only the effects of *AMY1* CNV on salivary amylase protein expression and amylase enzyme activity but also whether there is any evidence for underlying difference between the common haplotypes containing odd numbers of *AMY1* and even copy number haplotypes.

**Results:**

*AMY1* copy number was significantly correlated with the variation observed in salivary amylase production (11.7% of variance, *P* < 0.0005) and enzyme activity (13.6% of variance, *P* < 0.0005) but did not explain the majority of observed variation between individuals. *AMY1*-odd and *AMY1*-even haplotypes showed a different relationship between copy number and expression levels, but the difference was not statistically significant (*P* = 0.052).

**Conclusions:**

Production of salivary amylase is correlated with *AMY1* CNV, but the majority of interindividual variation comes from other sources. Long-range haplotype structure may affect expression, but this was not significant in our data.

**Electronic supplementary material:**

The online version of this article (doi:10.1186/s40246-017-0097-3) contains supplementary material, which is available to authorized users.

## Introduction

The enzyme amylase plays a major role in starch hydrolysis, which begins in the oral cavity and continues into the stomach and then small intestine. Amylase is the most abundant protein in saliva, accounting for at least 50% of salivary protein [1], but the quantity and enzyme activity of salivary amylase varies greatly among individuals. This variation in amylase production could be attributable to a number of factors including environmental factors, such as stress [2] and circadian rhythms [3], oral health [4] and the genetic background of an individual’s amylase gene cluster. Whilst it has been suggested that quantitative variation in amylase protein patterns does not always reflect variation in the amylase gene cluster [5], some studies have shown a relationship between the observed copy number variation (CNV) at the salivary amylase gene (*AMY1*) and an increased level of amylase protein expression [6, 7]. Perry et al. [7], using immunoblotting to investigate amylase protein levels, identified a significant positive correlation (*R* = 0.59) between CNV at *AMY1* and levels of amylase protein in saliva. Mandel and colleagues [6], also using immunoblotting, observed a similar correlation (*R* = 0.50) with copy number at *AMY1* and amylase protein levels as well as a correlation (*R* = 0.52) between CNV at *AMY1* and salivary enzyme activity. These results suggest that approximately 20–35% of the variance in salivary amylase expression can be attributed to variation in *AMY1* copy number.

The human amylase genes form a cluster on chromosome 1 which contains both the salivary (*AMY1*) and pancreatic (*AMY2*) amylase genes, both of which vary in copy number [5, 8, 9]. The CNV at *AMY1* has an observed range of 2–18 copies per person [7, 10–12] and an average of 6 copies per person, whilst the CNV at *AMY2* has an observed range of 2–12 copies per person and an average of 4 copies per person. The amylase gene cluster is highly structured [13–15], with a correlation between the CNV at *AMY1* and the CNV at *AMY2* [10, 12]. Recent observations have identified a fundamental difference in the underlying genomic structure across the amylase gene cluster between majority haplotypes containing an odd number of copies of *AMY1* and one copy each of *AMY2A* and *AMY2B*, and less common variant haplotypes containing even copy number haplotypes of *AMY1* and deletions or duplications of *AMY2* genes. Consequently, the majority of individuals (60–70%), with two *AMY1*-odd haplotypes, have an even copy number of *AMY1* and no CNV of *AMY2*, whereas those individuals with an odd copy number of *AMY1* (usually heterozygous for an *AMY1*-even haplotype) also display CNV of *AMY2* [10, 12].

Previous studies investigating the relationship between CNV at *AMY1* and salivary amylase protein expression have used qPCR to measure copy number. However, qPCR measurement of *AMY1* has since been shown to be subject to systematic error and, in one study, consistently underestimated the *AMY1* copy number [7, 10]. We aimed to re-evaluate the relationship between CNV at *AMY1* and salivary amylase protein expression using alternative copy number measurement methods that have been shown to be precise and reproducible [10]. Our experimental plan was designed to measure both the expression of salivary amylase total protein and amylase enzyme activity in saliva from a larger cohort of individuals than has been previously studied, in parallel with determination of copy number at *AMY1*. Knowledge of the haplotype structures also allows us to test whether all copies of *AMY1* are functionally equivalent or whether there is any evidence for context dependence of gene expression. Therefore, our aim is to explore the functional consequences of the multi-allelic copy variable gene *AMY1* on more (*N* = 119) samples than previously investigated and using novel methods of *AMY1* copy number measurement, capable of resolving single integer copy numbers.

## Results

### Variation in AMY1 and AMY2 copy numbers

**Fig. 1:**
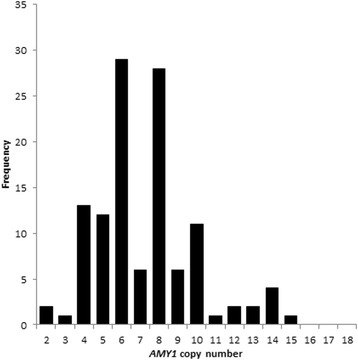
Distribution histogram of *AMY1* copy number in 119 unrelated UK samples; a clear majority have even copy numbers (89 out of 119)

Copy number measurement of both *AMY1* and *AMY2* was performed on all 119 independent UK samples (see “Methods”). The *AMY1* copy number distribution is shown in Fig. 1 and shows a predominance of even copy numbers (75%), with a range of 2–15 and a modal copy number of 6, consistent with prior studies of *AMY1* copy number [7, 10–12]. Variation in *AMY2* copy number was also observed with *AMY2A* copy variable in 24% of samples and *AMY2B* showing CNV in about 10% of samples (Table 1).

**Table 1:** CNV of *AMY2* in 119 UK samples studied

Copy number	*AMY2A*	*AMY2B*
0	0	0
1	14	0
2	91	107
3	13	12
4	1	0
TOTAL	119	119

### Correlation of AMY1 copy number with protein production and enzyme activity

We investigated both amylase protein levels and salivary amylase enzyme activity. Our data are consistent with previous studies in exhibiting considerable variation in protein expression [6, 7] and include some samples (across all copy numbers) with very low amounts of amylase protein (lowest value 0.48 mg/mL), as also detected by Mandel et al. [6].

**Fig. 2:**
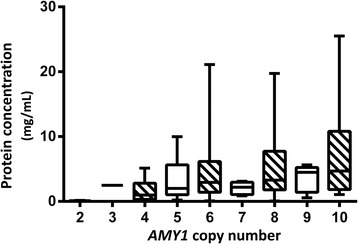
A *box* and *whiskers plot* of amylase protein concentration by *AMY1* copy number, with mean for all samples at a given copy number shown as a *black bar*, the standard deviation as the *box*, and *whiskers* showing the observed full range of data. Each contributing data point is the mean of three experimental replicates, and further details of biological replicates from the same subjects can be found in the “Methods”

**Fig. 3:**
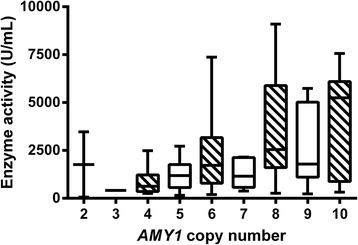
A *box* and *whiskers plot* of amylase enzyme activity by *AMY1* copy number, with mean of all samples at each copy number shown as a *black bar*, and *whiskers* showing the observed range of data. The *boxes* indicate the standard deviation at each copy number. Each data point used in the analysis is the mean of two experimental replicates, and further details of biological replicates from the same subjects can be found in the “Methods”

The raw data for total protein (antigen) concentration (Fig. 2) and for enzyme activity (Fig. 3) did not show a strong relationship with copy number, and the residuals of the regression are far from normally distributed (Additional file 1: Figure S2). A linear regression was performed using log_10_ of protein and of enzyme activity giving residuals that follow a normal distribution (Additional file 1: Figure S2) and satisfy other assumptions of linear regression modelling, and therefore, all further analyses were performed with the transformed data.

A significant correlation was observed between *AMY1* copy number and amylase protein (*R* = 0.342) (*P* < 0.0005). Our data suggests that *AMY1* copy number accounts for 11.7% of the variation observed in salivary amylase protein levels, much less than previous reports of 35% from a study of 50 European American individuals [7], and 25% in a study of 62 individuals of unspecified ancestry [6]. Our observation suggests that the CNV at *AMY1* plays much less of a role in the variation of salivary amylase protein levels than previously proposed. Furthermore, the great spread of observed levels of amylase protein production suggests that the genetic contribution of the CNV is not simply proportional to protein production.

A similar relationship was observed with salivary amylase enzyme activity. A significant correlation was observed between *AMY1* copy number and enzyme activity (*R* = 0.369) (*P* < 0.0005). Again, our data suggest that copy number plays less of a role than previously reported, with copy number accounting for 13.6% of the variation in enzyme activity observed in our study, rather than 27% as previously suggested [6]. A similar observation was reported in a Chinese population (*n* = 92) which found the gene copy number provided 12.2% of the observed salivary enzyme activity variation [16]. However, direct comparison between these studies is not straightforward, as Chinese populations do have a distinct *AMY1* distribution to Europeans [10, 12].

As our copy number measurement system is accurate enough to assign single integers, we were able to investigate whether there are differences in gene expression between those individuals that have even *AMY1* copy numbers and those with odd. We fitted a logistic regression model to protein and copy number data and used the model to predict protein expression from copy number to examine whether the odd or even number status was associated with a systematic difference in the relationship. This analysis gave marginally non-significant evidence (*P*= 0.052) for a difference between the regressions of log protein with even (*R* = 0.168) and odd (*R* = 0.017) copy numbers (Additional file 1: Figure S3), suggesting that the relationship between copy number and protein may be different for the odd and even copy numbers.

## Discussion

This is only the second study to investigate both protein levels and enzyme activity in the same samples. There is a highly significant correlation between the two measures (*R* = 0.66; *P* < 0.0001) (Additional file 1: Figure S4); whilst this is not a strong relationship, it is consistent with previous observations between these two measures (*R* = 0.61) [6]. This observation does suggest that for a particular quantity of amylase protein, there are variations in measurable enzyme activity, and supports the proposal that the enzymatic functions of amylase may be affected by protein modifications or the formation of complexes [6, 17].

With the complex underlying structure at the amylase gene cluster, it is possible that longer-range structure, including the CNV at *AMY2*, may influence *AMY1* expression, but there was no significant correlation observed between CNV at either *AMY2A* or *AMY2B* with either salivary amylase protein production or enzyme activity.

## Conclusions

To re-assess the relationship between copy number and salivary amylase protein expression and activity, our work used a more accurate and precise *AMY1* copy number measurement method than the qPCR methods previously employed. Our previous work demonstrated the reliability of PRT-based methods and the susceptibility of qPCR methods to measurement error [10]. Our data clearly show that copy number plays much less of a role in salivary amylase expression and activity than has been previously suggested, and that it is not possible to predict an individual’s salivary amylase concentration or enzyme activity solely from their copy number, with implications for studies of the effects of *AMY1* copy number variation, such as with diet. It is interesting to speculate on possible reasons for the differences between our results and those of other researchers. In addition to the improved methodology for copy number measurement, there may also have been differences in the sampling regime for saliva; in our work, we specified the method and time of collection but did not examine or standardise other factors, such as the timing relative to meals. Because of the fundamental structural difference between haplotypes containing odd or even numbers of copies, we wanted to test the possibility that odd or even number haplotypes might have different relationships between copy number and gene expression. Our data neither confirm nor exclude functional differences that arise from the underlying genomic structure across the amylase region between odd and even copy number haplotypes. Further studies would be needed to support the idea, but it does remain possible that the longer-range genomic structure, in addition to *AMY1* copy number itself, may have a role in determining variation in gene expression.

## Methods

### Study population

Our analysis utilised 120 independent volunteers from the University of Nottingham staff and student body, with 10 randomly selected to provide repeat samples. The blood, for DNA extraction, and saliva, for salivary amylase analysis, were taken with full consent from individuals and under local ethical approval (University of Nottingham Medical School Ethics Committee approval reference number BT10/02/2010). All samples were of the UK origin with no known clinical phenotype. DNA was extracted using isolated lymphocytes and a standard ‘salting out’ method for protein removal followed by phenol-chloroform extraction. DNA concentration was measured using a NanoDrop spectrophotometer, and DNA purity was assessed from the 260:280 nm absorbance ratio. All samples were diluted to a working concentration of 10 ng/mL. DNA was successfully extracted for all samples, except one, giving a total sample size of 119 independent individuals.

### Sample preparation

The saliva samples were collected from each volunteer at approximately 9.30 am (+/−10 min). The volunteers chewed on a 4 cm piece of parafilm for 30 s to allow saliva to be produced and then collected into a 15 ml sterile polypropylene container. The tubes were centrifuged at 13 rpm for 5 min to remove any solids from the suspension, and the remaining saliva was stored at −80 °C. For genotyping, 20 mL of the whole blood was taken from each volunteer from which genomic DNA was isolated and stored at −80 °C.

### Measurement of AMY1 and AMY2 copy numbers

The copy number of *AMY1* was measured from genomic DNA using a paralogue ratio test (PRT) in combination with a TATC microsatellite assay, as previously described [10]. *AMY2* copy number was measured using an *AMY2A*:*AMY2B* ratio assay, an *AMY2A*:*AMY2A* pseudogene ratio assay and an *AMY2A/2B* duplication junction assay, as previously described [10].

PRT PCR reactions were performed using previously described primers PRT_ref12 [10] that amplify from each copy of *AMY1* and from a reference locus at hg19 chr12:9,867,565–9,867,813. PCR products were mixed with 10 μl HiDi formamide with ROX-500 marker (Applied Biosystems, Warrington, UK), and subsequent fragment analysis was carried out by electrophoresis on an ABI3130xl 36 cm capillary using POP-7 polymer with an injection time of 30 s at 1 kV. GeneMapper software (Applied Biosystems, Warrington, UK) was used to extract the peak areas for the PRT and calculate the ratio of test (244 bp) to reference (249 bp) products. Copy number values were calculated by calibrating the ratios using HapMap CEU samples [NA11930 with *AMY1* copy number (CN) = 2; NA06993 with CN = 6; NA10852 with CN = 6; NA10835 with CN = 8; NA12248 with CN = 8; NA11931 with CN = 8; NA11993 with CN = 10 and NA07347 with CN = 11], which were included in every experiment in duplicate.

For further confirmation of *AMY1* gene copy number, a TATC microsatellite PCR was performed for each sample [10]. A single PCR reaction was performed and the products were mixed with 10 μl HiDi formamide with ROX-500 marker (Applied Biosystems, Warrington, UK), and fragment analysis was carried out by electrophoresis on an ABI3130xl 36 cm capillary using POP-7 polymer with an injection time of 30 s at 1 kV. GeneMapper software (Applied Biosystems, Warrington, UK) was used to extract the peak areas.

The ratio of *AMY2A* copy number to *AMY2B* copy number and the ratio of *AMY2A* copy number to *AMY2A* pseudogene copy number were measured as previously described [10]. One microliter of PCR products from both assays were mixed and added to 10 μl HiDi formamide with ROX-500 marker (Applied Biosystems, Warrington, UK), and fragment analysis was carried out by electrophoresis on an ABI3130xl 36 cm capillary using POP-7 polymer, injecting at 1 kV for 10 s. GeneMapper software (Applied Biosystems, Warrington, UK) was used to extract the peak areas and calculate the ratio of *AMY2A* (163 bp) to *AMY2B* (167 bp) and the ratio of *AMY2A* (197 bp) to *AMY2A* pseudogene (232 bp).

The *AMY2A/2B* duplication junction assay is a three-primer assay producing PCR amplicons of 424 bp in all samples and a specific 323 bp only from the duplication junction. The products were visualised on a 2% (*w*/*v*) agarose gel, as previously described [10].

### Measurement of amylase protein

The concentration of amylase protein antigen present in the saliva was measured using a sandwich amylase ELISA with 1 μg/mL of anti-salivary amylase antibody (Abcam, Cambridge, UK) and 100 μg/mL of biotinylated detection antibody (Biorbyt, Cambridge, UK). Assays were performed using serial dilutions (1:5) of natural human salivary amylase protein of known concentration (200 μg/mL) (Sigma-Aldrich, Gillingham, Dorset, UK) to generate a standard curve. Assays were performed in duplicate for each unknown sample, and the standard curve was measured in triplicate.

### Measurement of amylase enzyme activity

The amylase enzyme activity within saliva was measured using the EnzCheck® *Ultra* Amylase Assay Kit (Invitrogen, ThermoFisher, Paisley, UK) according to manufacturer’s instructions. The saliva samples were added to the substrate solution, vortexed, and the fluorescence of the samples was measured after 10 min incubation at room temperature. Assays were performed using serial dilutions (1:5) of natural salivary amylase protein of known concentration (200 μg/mL) (Sigma-Aldrich, Gillingham, Dorset, UK) to generate a standard curve. Assays were performed in duplicate for each unknown sample, and the standard curve was measured in triplicate.

In order to deduce the variation in concentration for protein expression and enzyme activity within each individual, the repeat samples from an initial cohort of 10 individuals were investigated on four separate occasions (Additional file 1: Figure S1). These 10 repeat samples comprise samples with 2 (×1), 4 (×1), 6 (×4), 8 (×2) and 9 (×2) copies of *AMY1*. Analysis of repeat measures found that neither of the within-subject factors (time and measurement) was significant for measurement of salivary amylase protein expression or enzyme activity, and therefore, a single time point is suitable for comparing measurements of salivary amylase protein and enzyme activity between individuals.

### Bradford assay for measurement of total protein concentration

The total protein concentration in the saliva samples was measured using Bradford Reagent (Sigma-Aldrich, Gillingham, Dorset, UK) according to manufacturer’s instructions. Assays were performed using serial dilutions (1:2) of bovine serum albumin (BSA) protein of known concentration (1.4 mg/mL) (Sigma-Aldrich, Gillingham, Dorset, UK) to generate a standard curve. Assays were performed in duplicate for each unknown sample, and the standard curve measured in triplicate.

### Statistical analysis

Correlations between groups of copy number data and either protein expression or enzyme activity were assessed using logistic regression in SPSS V22 (IBM, Armonk, New York, USA), and figures were drawn with the software GraphPad Prism (GraphPad Software, Inc., La Jolla, CA, USA). The repeat measures were analysed in SPSS using a general linear model with repeated measures, with the within-subject factors defined as time and measurement and the between-subject factor defined as copy number.

## Additional files

Additional file 1: Additional Figures for Carpenter et al., “Copy number variation of human AMY1 is a minor contributor to variation in salivary amylase expression and activity”. **Figure S1.** Graphs for the time trial samples illustrating the overall variation observed for each sample from the four separate time points. The samples are sorted by copy number for amylase protein concentration (A) and enzyme activity (B). **Figure S2.** Normal P-P plots of the residuals from logistic regression analysis for (A) amylase protein concentration, (B) Log10 protein concentration, (C) amylase enzyme activity and (D) Log10 enzyme activity. **Figure S3.** Graph of the residuals from logistic regression between log10 protein (LogProtein) with odd (blue circles) and even (green circles) AMY1 copy numbers shown separately. **Figure S4.** Correlation between salivary amylase protein levels (mg/mL) and amylase enzyme activity (U/mL). (PDF 425 kb)
Additional file 2: Dataset (XLSX 22 kb)

## Abbreviations

BSA: Bovine serum albumin
CN: Copy number
CNV: Copy number variation/variant
ELISA: Enzyme-linked immunosorbent assay
HiDi: Highly deionised (formamide)
