# Supplementary material for: Copy number variation of human AMY1 is a minor contributor to variation in salivary amylase expression and activity
Source: Hum Genomics. 2017 Feb 20;11:2. doi: 10.1186/s40246-017-0097-3 (PMC5319014; doi:10.1186/s40246-017-0097-3)
Supplement: Additional file 1: — Additional Figures for Carpenter et al., “Copy number variation of human AMY1 is a minor contributor to variation in salivary amylase expression and activity”. Figure S1. Graphs for the time trial samples illustrating the overall variation observed for each sample from the four separate time points. The samples are sorted by copy number for amylase protein concentration (A) and enzyme activity (B). Figure S2. Normal P-P plots of the residuals from logistic regression analysis for (A) amylase protein concentration, (B) Log10 protein concentration, (C) amylase enzyme activity and (D) Log10 enzyme activity. Figure S3. Graph of the residuals from logistic regression between log10 protein (LogProtein) with odd (blue circles) and even (green circles) AMY1 copy numbers shown separately. Figure S4. Correlation between salivary amylase protein levels (mg/mL) and amylase enzyme activity (U/mL). (PDF 425 kb) [file 40246_2017_97_MOESM1_ESM.pdf]

Additional Figures for Carpenter *et al*., **“Copy number variation of human *AMY1* is a minor contributor to variation in salivary amylase expression and activity”.**

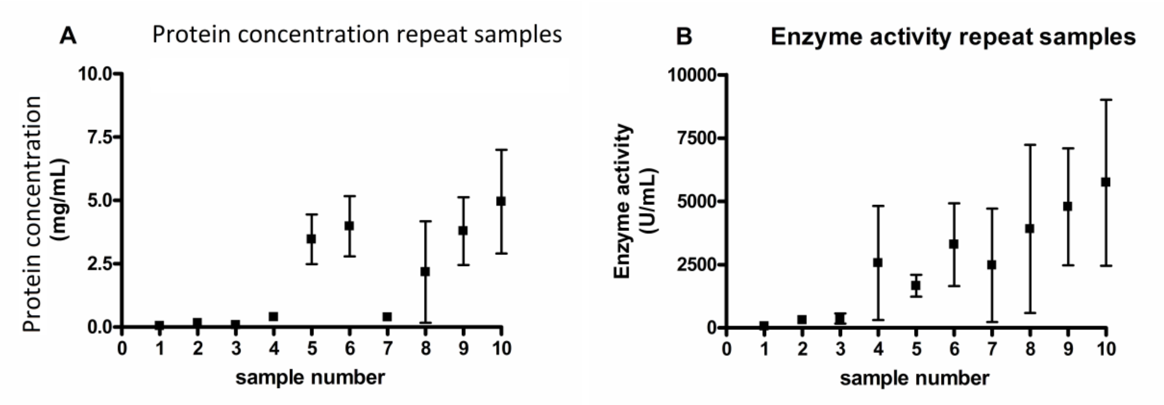

Additional Figure 1: Graphs for the time trial samples illustrating the overall variation observed for each sample from the four separate time points. The samples are sorted by copy number for amylase protein concentration (A) and enzyme activity (B).

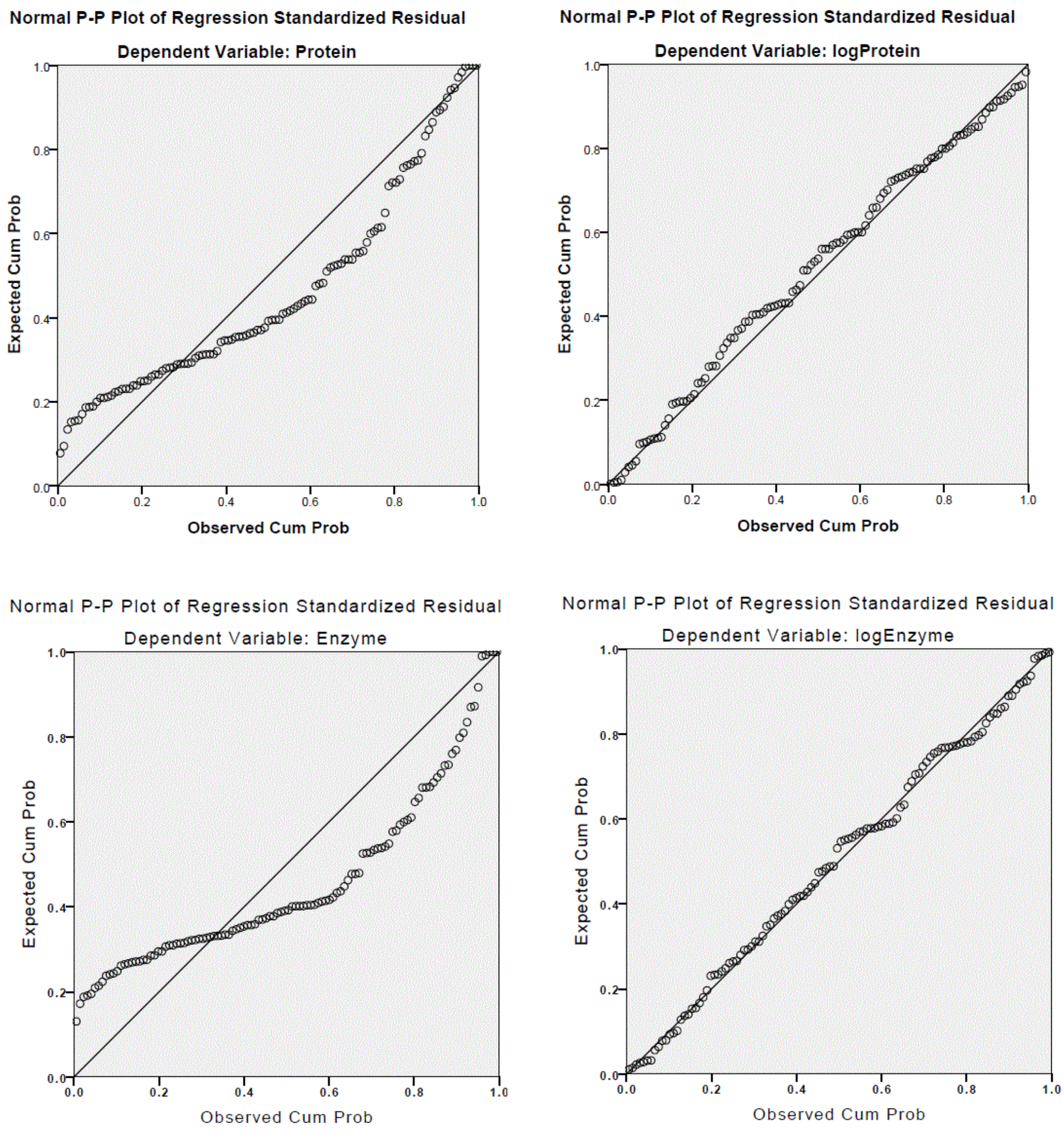

Additional Figure 2: Normal P-P plots of the residuals from logistic regression analysis for (A) amylase protein concentration, (B) Log_{10} protein concentration, (C) amylase enzyme activity and (D) Log_{10} enzyme activity.

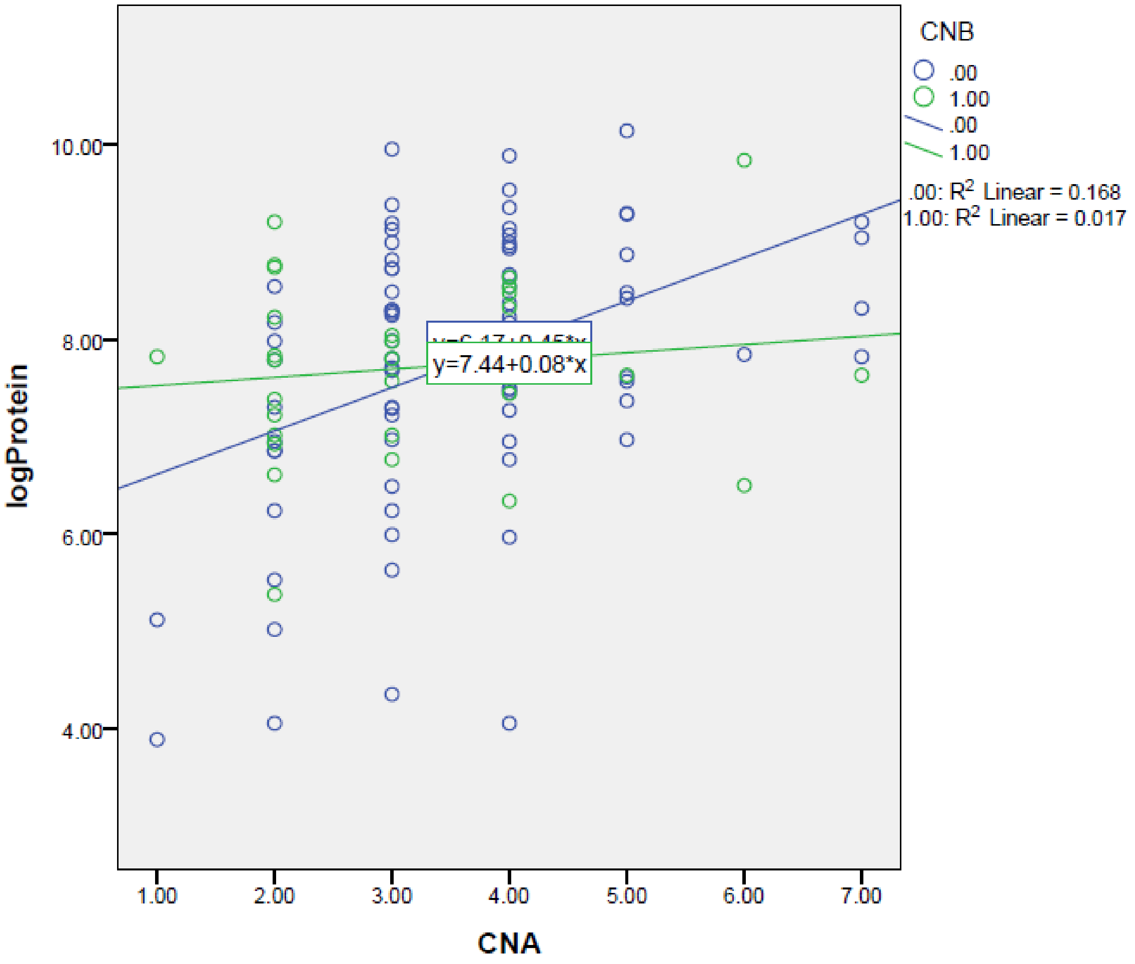

Additional Figure 3: Graph of the residuals from logistic regression between log_{10} protein (LogProtein) with odd (blue circles) and even (green circles) *AMY1* copy numbers shown separately.

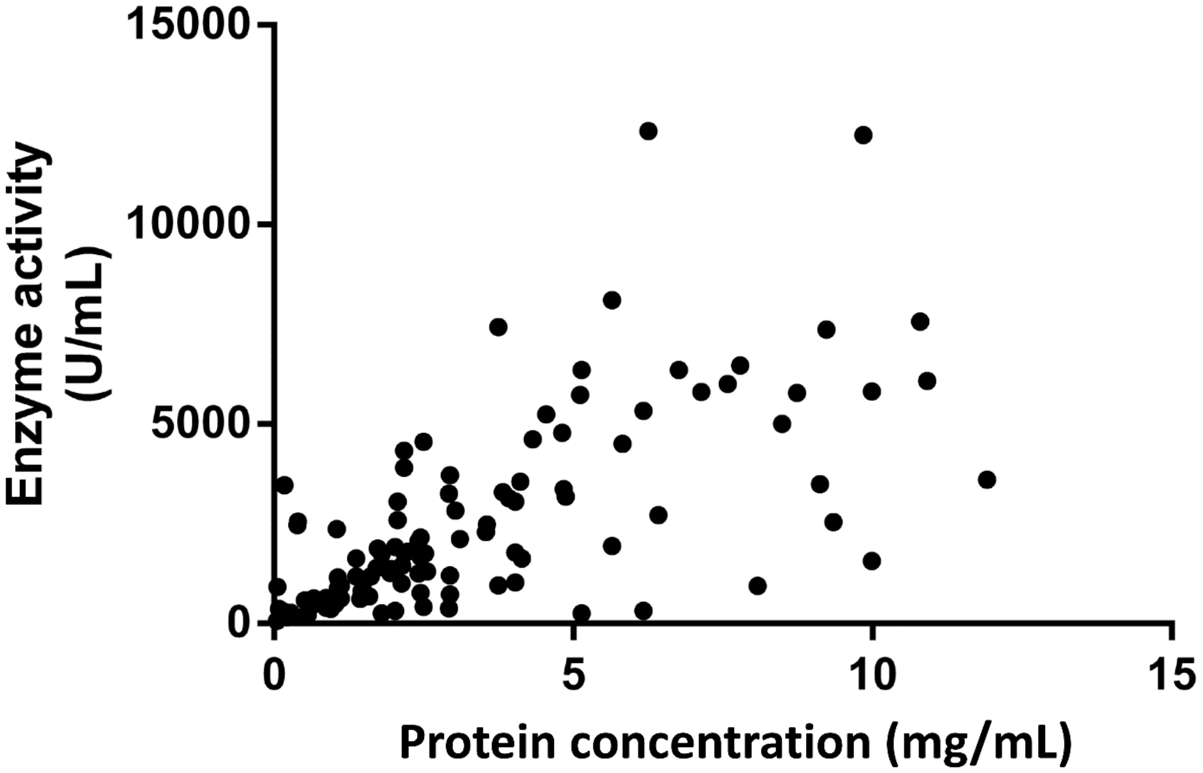

Additional Figure 4: Correlation between salivary amylase protein levels (mg/mL) and amylase enzyme activity (U/mL).
